# Delirium screening in critically ill patients: a systematic review and meta-analysis

**DOI:** 10.1186/cc10944

**Published:** 2012-03-20

**Authors:** A Serpa Neto, AP Nassar Júnior, SO Cardoso, JA Manetta, VG Pereira, DC Esposito, MC Damasceno, AJ Slooter

**Affiliations:** 1 ABC Medican School, Santo André, Brazil; 2 São Camilo Hospital, São Paulo, Brazil; 3 University Medical Center Utrecht, the Netherlands

## Introduction

Despite its frequency and impact, delirium in critically ill patients is poorly recognized. Our aim was to systematically review the accuracy of delirium screening instruments in critically ill patients.

## Methods

Systematic review and meta-analysis of publications between 1966 and 2011. The Medline and Embase databases were searched for studies on delirium in critically ill patients in ICUs, surgical wards or emergency rooms. The delirium screening tool had to be feasible in a clinical setting for use by a nonexpert. As the gold standard, delirium had to be diagnosed based on appropriate criteria by a delirium expert. The outcomes assessed were: sensitivity, specificity, likelihood ratios and summary receiver-operating characteristic (ROC) curves.

## Results

**Figure 1:**
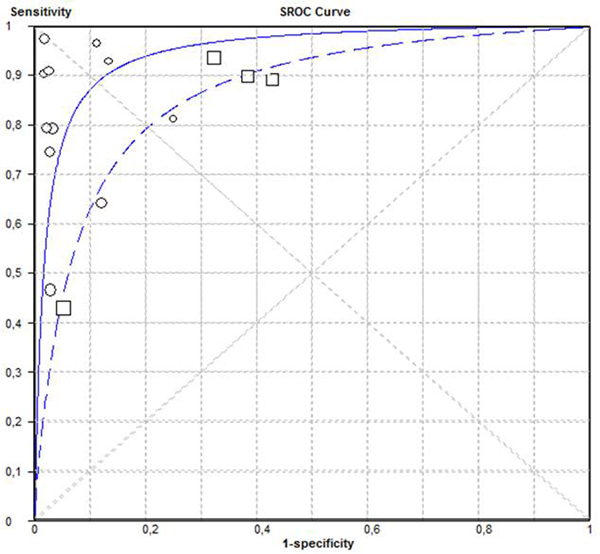
**ROC curve: CAM-ICU (solid line and circles) and ICDSC (dashed line and squares)**.

Fifteen studies covering 1,404 participants and five screening tools were included in the systematic review. The pooled sensitivities and specificities for CAM-ICU for detection of delirium in critically ill patients were 76.0% and 95.7% and for ICDSC were 74.4% and 75.2%, respectively. All but one study was performed in a research setting, and that one study suggested that, with routine use of CAM-ICU, one-half of the patients with delirium were not detected. See Figure 1.

## Conclusion

The CAM-ICU was the most specific bedside tool for assessment of delirium in critically ill patients. However, there was significant heterogeneity of the results. These findings were largely obtained in research settings, and the low sensitivity of the CAM-ICU in routine, daily practice may limit its use as a screening test.

